# Heavy Metal Ions Removal from Aqueous Solutions by Treated Ajwa Date Pits: Kinetic, Isotherm, and Thermodynamic Approach

**DOI:** 10.3390/polym14050914

**Published:** 2022-02-25

**Authors:** Mohammad Azam, Saikh Mohammad Wabaidur, Mohammad Rizwan Khan, Saud I. Al-Resayes, Mohammad Shahidul Islam

**Affiliations:** Department of Chemistry, College of Science, King Saud University, P.O. Box 2455, Riyadh 11451, Saudi Arabia; swabaidur@KSU.EDU.SA (S.M.W.); mrkhan@ksu.edu.sa (M.R.K.); sresayes@ksu.edu.sa (S.I.A.-R.); mislam@KSU.EDU.SA (M.S.I.)

**Keywords:** biosorbent, copper, adsorption, model studies, aqueous medium

## Abstract

In the current study we prepared cost-effective adsorbents based on ajwa date pits to remove Cu(II) ions from aqueous medium. Adsorbents were studied using scanning electron microscopy (SEM), FT-IR, and Brunauer-Emmett-Teller (BET) methods to characterize the surface functionalities, morphology, pore size, and particle size. The concentration of Cu(II) ions in the studied samples was determined by atomic adsorption spectrometry technique (AAS). Adsorption method was performed sequentially in a batch system followed by optimization by studying the numerous conditions, for instance the initial amounts of Cu(II) ions, dosages of the adsorbent, contact time, and pH of the solution. The ideal pH observed for maximum adsorption capacity was ~6.5. Langmuir and Freundlich isotherm models correctly predicted the investigation results, with the maximum monolayer adsorption capacities for Cu(II) ions at 328 K being 1428.57 mg/g (treated ajwa date pits, TADP) and 1111.1 mg/g for as produced ajwa date pits (ADP). It was revealed that TADP possess greater adsorption capability than ADP. Recovery investigations revealed that the saturated adsorbents eluted the maximum metal with 0.1 M HCl. Cu(II) ions adsorption was observed to be reduced by 80–89% after the second regeneration cycle. For the raw and chemically processed ajwa date pits adsorbent, the Langmuir model performed significantly better than the Freundlich model. The results demonstrated that the adsorbent made from ajwa date pits could be an economical and environmentally friendly alternative for removing Cu(II) ion pollutant from aqueous media.

## 1. Introduction

Heavy metals hazardous waste has currently become one of the most serious environmental issues [1,2]. Heavy metal contamination accumulates in the environment and is not naturally decomposable, posing a concern to human health [3,4]. Cu(II) ion is a heavy metal ion that is widely used in a variety of manufacturing industries, including fertilizer, paints, plating baths, and paper goods [5,6,7,8]. The discharges from these sources contain a significant amount of Cu(II) ions, which are discharged into the environment via watercourses and earth [7,8]. It eventually combines with the food chain to cause a variety of severe human ailments [9]. Long-term copper exposure is harmful to health and can cause several diseases such as diarrhoea, vomiting, central nervous system and, mucosal irritation, hepatic, gastrointestinal distress and, kidney and liver damage [10,11,12,13,14]. In addition, if a high quantity of copper salts is consumed, it forms severe copper toxicity in individuals, most likely because of redox cycling and the production of reactive oxygen species that harm deoxyribonucleic acid [15,16]. Regarding its exposure to human, the US Occupational Safety and Health Administration has labelled an allowable exposure limit for copper fumes and dusts in the workplace as a time-weighted average of 1 mg/m^3^ [17]. The National Institute for Occupational Safety and Health has fixed a suggested exposure limit of 1 mg/m^3^ as the time-weighted average. The immediate risk to life and health concentration is 100 mg/m^3^ [17]. Recently, the National Primary Drinking Water Regulations of the USEPA has also regulated the levels of Cu(II) in drinking water with MCL of 1.3 mg/L [14]. The Cu(II) ion has also been regulated by the National Primary Drinking Water Regulations of the United States Environmental Protection Agency (USEPA) in drinking water with a Maximum Contaminant Level (MCL) of 1.3 mg/L.

Several traditional techniques such as ion-exchange [18], oxidation [19], electrocoagulation [20], solvent extraction [21], chemical precipitation [22], ultrafiltration [23], adsorption [24], activated carbon [25], membrane separation [26], and evaporation [27] have been applied to eliminate heavy metal ions. Nonetheless, most of these techniques have some drawbacks such as high production cost, consumption of high energy, high volumes of waste, and challenging treatment systems. Although, the adsorbent based on activated carbon has great microporous features, including high adsorption capacity and high surface area, it has demonstrated its capability as an adsorbent for the heavy metals removal from wastewater [28]. However, the application of activated carbon is very costly, with moderately high operational overheads. In addition, there is a requirement of regeneration prior to each adsorption phase [25]. Among these procedures, adsorption has been the most extensively accepted process because of its simplicity, versatility, ecofriendly, and being highly cost effective [24]. However, for the present, there is a rising demand to discover effortlessly obtainable, highly efficient, and very cost effective adsorbents for the adsorption of Cu(II) ions from different matrices. Cashew nut shell [5], saw dust [29], spent grain [30], wheat straw [31], pine cone powder [32], sawdust [33], herbaceous peat [34], peanut hull [6] sugar beet pulp [35], and others [36,37,38,39] are examples of effective adsorbents to remove copper ions. Due to the great obtainability and low-price of these adsorbents a complex regeneration method is not needed, and this cheaper adsorption system has involved numerous researchers. Thus, in order to substitute the current marketable materials, we have chosen ajwa date pits as the adsorbent, because Saudi Arabia is the world’s third main producer of dates, and date pits are easily available and free of cost. In addition, the occurrence of numerous functional groups, for instance amino acid, carboxylic acid, phenols, ester, hydroxyl, and carboxylate can assist them as possible adsorbents for heavy metals removal from aqueous medium [40]. Therefore, we have developed adsorbents to remove Cu(II) ions from aqueous medium that are both cost effective and high-yielding, and are derived from a common waste product—ajwa date pits. Hydrogen peroxide (H_2_O_2_) treatment enhanced the surface functionalities (–OH) of the synthesized ADP powder. The raw and treated materials were characterized by different methods, for instance SEM, FT-IR and BET, certifying the excellent particle size, surface morphology, and adsorption capabilities. Several investigational parameters, including solution contact time, pH, starting concentration, and Cu(II) ion temperature, were also studied. Langmuir and Freundlich adsorption isotherms were applied to study the equilibrium modelling [36]. Thermodynamic and kinetic characteristics have been applied to assess the nature of the sorption method. In this study, we are using ajwa date pits as adsorbents for the first time to remove Cu(II) heavy metal ion from an aqueous solution.

## 2. Materials and Methods

### 2.1. Chemicals and Reagents

All the chemicals and reagents namely, copper(II) chloride (CuCl_2_), sodium hydroxide (NaOH), hydrochloric acid (HCl), sodium hydroxide (NaOH), and H_2_O_2_, 30% *v*/*v* were of analytical grade, and were obtained from Sigma-Aldrich (Steinheim, Germany). A stock solution of Cu(II) ion (1000 µg/mL) prepared in ultrapure water was purified using Milli–Q water purification system (Millipore Corporation, Bedford, NH, USA). For complete mixing, the solution was agitated for 5 min. The required Cu(II) ion test solutions were prepared by suitable successive dilutions of the stock solution. For calibration purposes the, concentration range of the Cu(II) ion ranged from 1 to 10 µg/mL. The pH of solutions was adjusted to the optimum value by NaOH/HCl.

### 2.2. Instrumentation

The amount of Cu(II) ion was identified by high-throughput atomic absorption spectrometry (AAS) (Thermo Scientific, Waltham, MA, USA). The point of zero charge was measured by means of the salt addition process (pHPZC). The surface morphology of the synthesized and treated date pit materials was determined using SEM (Jeol JSM 5400 L V, Tokyo, Japan). The surface functionalities present on the produced biosorbents were determined using FT-IR (Thermo Scientific Nicolet 6700, Waltham, MA, USA) with 1:100 wt ratio of KBr dilution equivalent to the 400 cm^−1^ and 4000 cm^−1^ wave numbers, obtained at 32 scans average value with a ±4 cm^−1^ resolution applied. Multifunctional X-ray diffractometer (XRD, Ultima IV, Rigaku, Charlestown, MA, USA) was used to determine the surface crystallinity and average particle size of the samples. The surface area and pore size of the samples were measured by BET surface area analyzer (Micromeritics-Gemini VII 2390 V1.03, Norcross City, GA, USA).

### 2.3. Biosorbent Preparation

**Figure 1:**
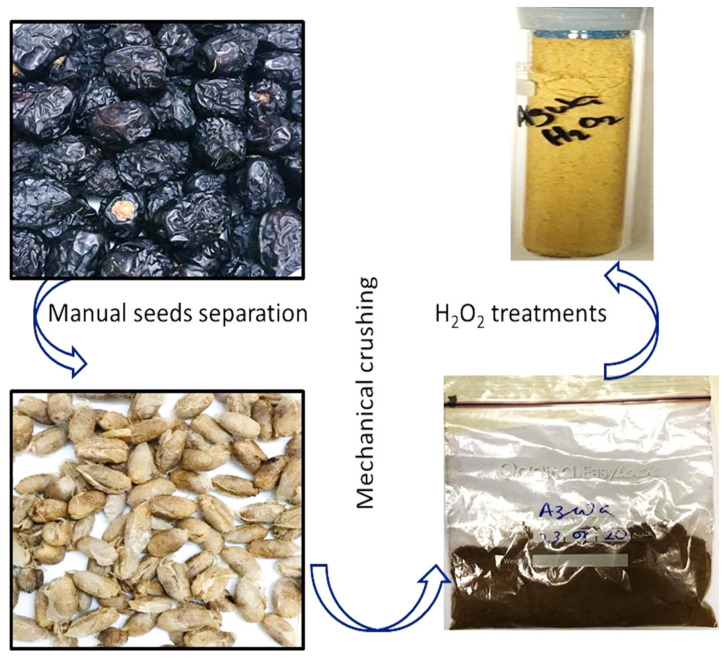
Ajwa dates, pits, and treated and untreated dates powders.

The ajwa dates (5 kg) were purchased from Kingdom Dates, Riyadh Saudi Arabia. The date pits were removed and thoroughly rinsed with MilliQ water to exclude any remnants of edible portion and dust materials, then sun dried for many days, and then dried in an oven for 2 h at 80 °C. The date pits were mechanically crushed and processed in a ball mill, homogenized, and sieved at 120 μm (Figure 1) [39,41].

To establish the negative functionalities (-OH) onto the surface of the ADP adsorbent, the prepared ADP adsorbents were treated with 200 mL of 30% H_2_O_2_ (*v*/*v*). Treatment with H_2_O_2_ also offers advantages to decompose organic parts, reduction of biomass resistance, and to avert the ADP cellulose degradation [41]. To perform the treatment procedure, ajwa date powder (10 g) was taken out in a beaker (100 mL) followed by the addition of the H_2_O_2_ solution (100 mL). The solution was then thoroughly mixed overnight using a magnetic stirrer. The Buckner funnel was used to filter the sample suspension with a vacuum pump, which was then washed with MilliQ water to eliminate any remaining content of hydrogen peroxide, and dried in an oven overnight. The samples were then allowed to cool to room temperature and bottled in transparent glass vials for further analysis.

### 2.4. Adsorption and Desorption Studies

Batch adsorption tests were carried out to eliminate Cu(II) ions from aqueous media. ADP powder (0.05 g) was weighed in a cleaned Erlenmeyer flask (100 mL) and Cu(II) (100 mg/L, C0) solution was added to the flask, which was shaken overnight at 100 rpm on a shaker. The solution mixture was separated and kept in a cold, dry environment. The AAS method was employed to quantify the amounts of Cu(II) ion, *C_e_* after the solution reached equilibrium.

At the time of equilibration time (*q_e_*) and arbitrary time *t* (*q_t_*), the adsorption (%) and Cu(II) ion removing property of the adsorbents were measured, as described in a previous study [41]:

(1) $$\;q\;\left(\%\right)=\frac{\left(C_{o}-C_{e}\right)}{C_{o}}\times 100,$$

where *q* is the adsorption, *C_o_* is the initial concentration, and *C_e_* is the equilibrium concentration.

(2) $$q_{e}=\left(C_{o}-C_{e}\right)\times \frac{V}{m},$$

where *q_e_* is the adsorption capacity at equilibrium, *C_o_* is the initial concentration, *C_e_* is the equilibrium concentration, V is the volume (L), and m is the mass of adsorbent (g).

(3) $$q_{t}=\left(C_{o}-C_{t}\right)\times \frac{V}{m},$$

where *q_t_* is the adsorption capacity at time *t* concentration (µg/mL) of Cu(II) solution, *C_o_* is the initial concentration, *C_t_* is the concentration at time *t*, and *V* is the volume (L), *m* is the mass of adsorbent (g). The adsorption capacities of (qe) and (qt) were measured in milligrams per gram.

For the pH investigations, at a concentration of 25 mg/L, the initial pH (pHi) of Cu(II) ions was changed from 2 to 10. At concentration 20–100 mg/L and temperatures 293–323 K, the effects of Cu(II) ion initial concentration (Co) on adsorption were tested.

Contact time (t) of Cu(II) ion adsorption was studied at Co: 25 mg/L for periods ranging from 2 min to 24 h.

Cu(II) ion solutions (50 mL; 25 mg/L) were saturated for 24 h with the adsorbents (0.05 g) for desorption studies. The saturated adsorbents were moderately washed with MilliQ water to eliminate the un-adsorbed remnants of treated Cu(II) ions. The Cu(II) ion was then eluted from the saturated adsorbent using a variety of eluents, including CH_3_COOH, H_2_SO_4_, HNO_3_, NaOH, and HCl solutions (50 mL, 0.1 mol/L). The percentage of desorption was calculated using the following amount of Cu(II) ions in the eluent:

% Desorption = (Desorbed amount of Cu(II) ions/adsorbed amount of Cu(II) ions on adsorbent) × 100

### 2.5. Thermodynamic Studies of Cu(II) Ions Adsorption

The thermodynamic conditions, which define adsorption spontaneity, are extremely important in adsorption investigations. The spontaneity of the adsorption process is demonstrated by a negative Gibb’s free energy change (ΔG°) at a known temperature. Equation (4) was used to calculate ΔG°

Δ*G*° = − RT lnKa. (4)

Equation (5) was used to calculate the values for enthalpy change (ΔH°) and entropy change (ΔS°).

Δ*G*° = Δ*H*° − *T*Δ*S*°, (5)

where R, T, and Ka represent the gas constant (8.314 J/mol K), temperature (K), and Langmuir constant [42], respectively.

## 3. Results and Discussion

### 3.1. Characterization

#### 3.1.1. Surface and Pore Size Analysis of Adsorbents

**Figure 2:**
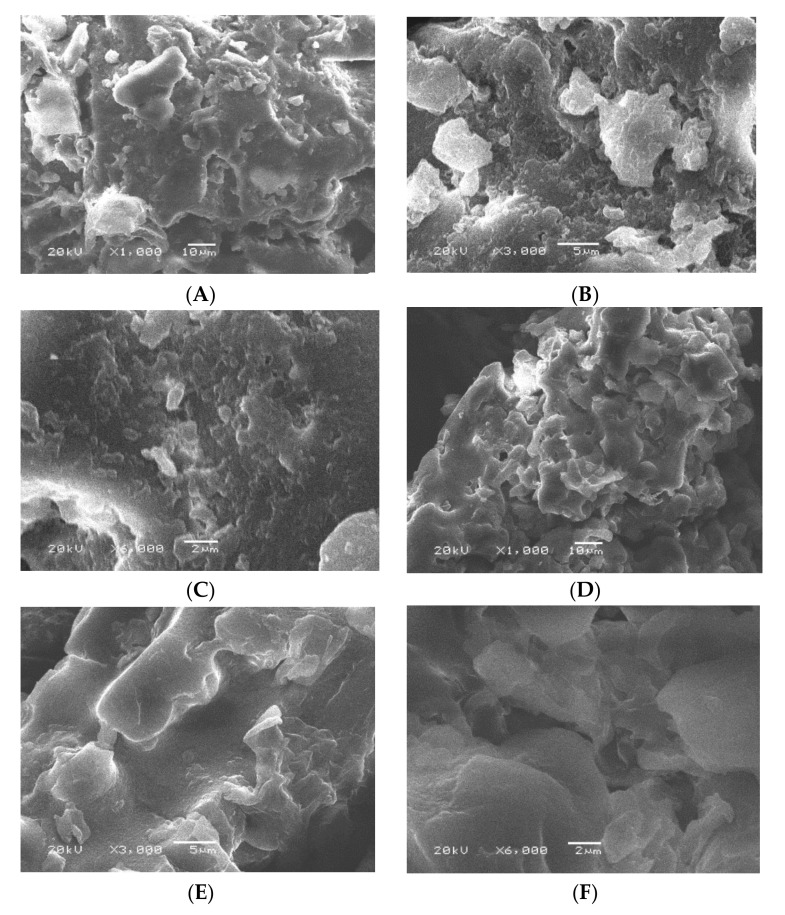
SEM images of ADP (**A**–**C**) and TADP (**D**–**F**) at various magnification: 1000× and 10 μm; 3000× and 5 μm: and 6000× magnification with 2 μm diameter.

The morphology and physical properties of the adsorbent surfaces of the prepared ADP and TADP adsorbents were evaluated using SEM. The SEM images of ADP and TADP are shown in Figure 2B,C. There are numerous fragmented particles that resemble spots, which can be seen in the SEM images of ADP (Figure 2A–C), showing the porous character of the adsorbents appropriate for target metal adsorption [39]. However, Scanning Electron Microscopic images of TADP discovered that the TADP causes the formation of a larger porosity surface, when compared to the raw material (Figure 2D–F).

The EDX analysis of the adsorbents was investigated to evaluate the composition of the active components presents in the adsorbents. The results indicate that the adsorbents are comprised of carbon as the major element (42 to 45%) and other negative heteroatoms such as oxygen (O_2_) and nitrogen (N_2_) with a relatively lower percentage (21 to 35%) compared to carbon (Table 1). It is obvious that the H_2_O_2_ treatment of ADP adsorbent shows higher contents of O_2_ as expected (35.8%), which indicates successful modification of the ADP surface into negatively charged hydroxyl functionalities (Table 1).

**Figure 3:**
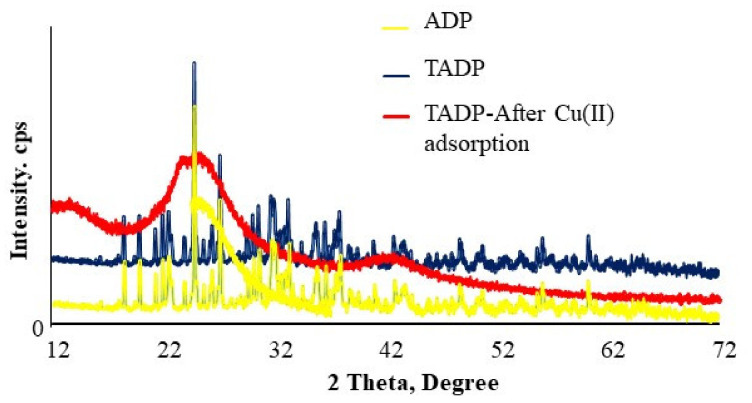
XRD analysis of ADP, TADP, and TADP after Cu(II) adsorption.

The powder X-ray diffraction analysis for the ADP and treated adsorbents (TADP) was carried out and displayed in Figure 3. The sharp peaks in the diffractograms indicates the crystalline nature of the prepared adsorbents, which were converted to wide peaks for Cu(II) ions saturated materials. This is likely due to the adsorption of metal ions on the surface of the materials, which caused the materials to change from crystalline to amorphous. The average crystallite size was also calculated by Scherrer’s equation and was determined to be 27 nm.

**Figure 4:**
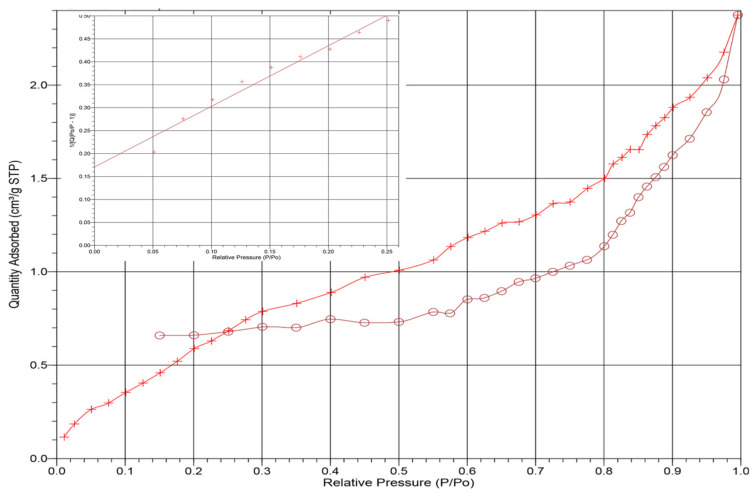
ADP linear isotherm plot with nitrogen adsorption plot (inset).

The BET surface areas for both ADP and TADP were determined to find the pore size of the materials (Figure 4). The surface area of TADP was found to be amplified from 2.47 to 8.48 m^2^/g. The changes in pore areas show that during the H_2_O_2_ treatment of the ADP materials, increased porosity develops, resulting in higher adsorption efficiencies [41]. The adsorption average pore widths were also determined for ADP and TADP and were found to be in the range of 6–12 nm.

#### 3.1.2. FTIR

**Figure 5:**
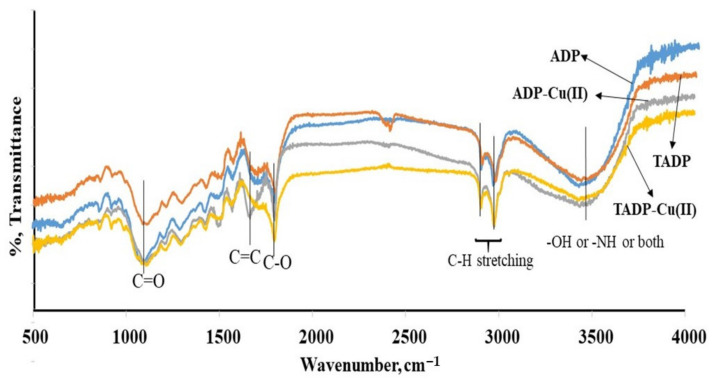
FT-IR analysis of ADP, ADP-Cu(II) ions, TADP, and TADP-Cu(II) ions (experimental conditions: initial conc. 25 mg/L; adsorbent weight 0.005 g; volume 0.05 L; temperature 25 °C; contact time 5 h; shaker speed: 100 rpm).

FTIR analysis was carried out to identify the surface functionalities and adsorption mechanism for ADP and TADP adsorbents before and after the adsorption Cu(II) ions (Figure 5). FTIR analysis helps to identify the functional groups as well as the bindings of the analyte with the adsorbents. The adsorbents materials are the lignocellulosic and are made up of lignin, cellulose, hemicellulose, and protein. Hydroxyl, ether, and carbonyl types oxygen-rich functionalities are abundant in both cellulosic and hemicellulose materials, which are mainly responsible for binding the metal ion with the adsorbent materials [41]. A broad peak in the FTIR spectra of both adsorbents at 3238–3570 cm^−1^ range, is attributed to the presence of the characteristic lignocellulosic peaks of -OH, -NH, or both -OH and NH_2_ [35].

The stretching vibrations of aliphatic C-H functionalities are responsible for the absorbance peaks of 2927 and 2850 cm^−1^. The occurrence of unconjugated carbonyl (C=O), imine (C=C) and C-O functionalities is indicated by vibrations at 1745, 1609, and 1045 cm^−1^, respectively. The presence of small peaks in the spectral area between the C-O and C=C, corresponds to bending peaks of -CH_3_ group [34,36]. The band at 514–708 cm^−1^ proved the existence of –OH polysaccharide groups. The number of small bands between the region 1410–1452 cm^−1^ appeared, owing to the presence of stretching vibrations of carboxylates (C–O and phenolic). Carboxylic acid and C-O-C vibrations were assigned to the bands appearing between 1210 and 1360 cm^−1^ and 1008 and 1159 cm^−1^, respectively.

The FTIR spectra of Cu(II) adsorbed ADP and TADP were also analyzed, and a minor shift in peaks (to higher wavenumbers) was observed at 3246–3580 cm^−1^ range, 1745 cm^−1^, and 1045 cm^−1^ as well as slight increases in those peak intensities, indicating the Cu(II) adsorption on adsorbents. The participation of the appropriate moieties (-OH, -NH, C-O, and C=O) in the adsorption of target metal ions is confirmed by small shifts and increases in peak intensities of such peaks. Furthermore, these findings reveal that the increased electronegativity of heteroatoms and the lone pair of electrons of the C=O, C-O, and –OH or NH functional groups are mostly liable for the coordination binding and electrostatic interactions that bind Cu(II) ions to the adsorbent surface [43].

### 3.2. Adsorption Properties

#### 3.2.1. Effect of pH of the Solution

**Figure 6:**
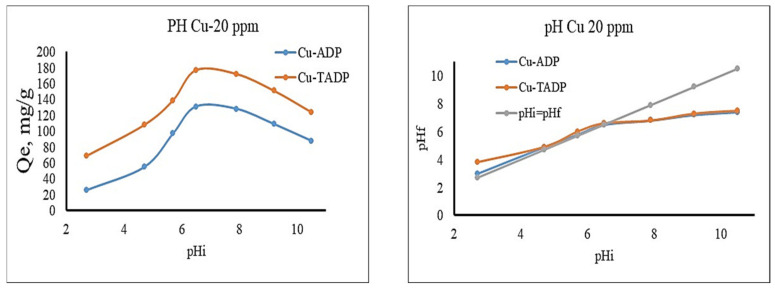
Adsorption capacity (qe) as a function of initial pH (pHi) and pHi versus pHf (final pH) plot for Cu(II) adsorption onto ADP and TADP. (Optimal conditions: initial concentration 25 mg/L; adsorbent weight 0.005 g; volume 0.05 L; temperature 25 °C; contact time 5 h; shaker speed 100 rpm).

Cu(II) adsorption on ADP and TADP was studied for initial pH (pH_i_) values ranging from 2.7 to 10.5. pH values higher than this range was omitted to avoid metal precipitation [41]. Because the Cu(II) metal ion was discovered to be cationic in the solutions phase, adsorption competition between the proton (H+) and metal ions is more likely at lower pH. This may be responsible for lower adsorption of the metal at highly acidic environments (Figure 6). Cu(II) ion adsorption capabilities rapidly increased after initial slowing, with maximum adsorption capacities of 131 mg/g and 177 mg/g for ADP and TADP at pH 6.5, respectively. The adsorption capacities of both adsorbents steadily decreased upon increasing the pH of the adsorption media (Figure 6).

Cu(II) ion adsorption was highest at pH 6.5, which was higher than the point of zero charges (pH_PZC_), and pH 6.4 for both ADP and TADP (Figure 6). The pH_PZC_ value becomes zero at pH 6.4, confirming the neutral surface of the adsorbent materials. The surfaces of the adsorbents become positively and negatively charged, respectively, below and above this pHpzc, suggesting that the surfaces of TMDP and TSDP are more likely too protonated under acidic conditions to prevent metal ion adsorption.

Furthermore, as the pH increases, protonation decreases, resulting in a decrease in surface positivity and an enhancement in Cu(II) ion binding over the ADP and TADP surfaces. Above pH 6.5, a drop in the pH graph was observed (Figure 6), indicating that the adsorbents surface was neutralized in combination with the adsorption equilibrium.

#### 3.2.2. Effect of Initial Concentration and Temperature

**Figure 7:**
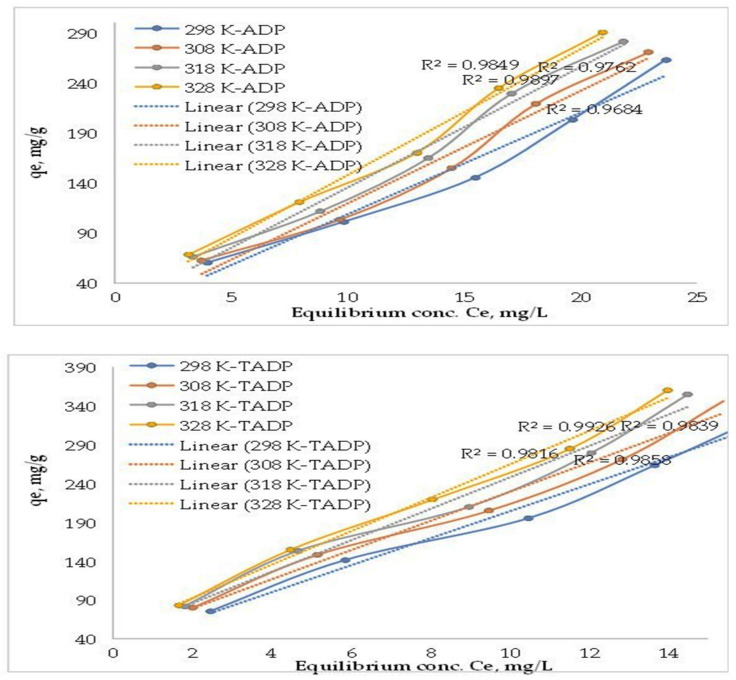
Effect of initial metal concentration and temperature (experimental conditions: initial conc: 25 mg/L; adsorbent weight: 0.005 g; volume: 0.05 L; temperature: 25 °C; contact time: 5 h; shaker speed: 100 rpm).

To determine the corresponding equilibrium isotherms, a different concentration of Cu(II) adsorption ranging from 10 to 50 mg/L on both ADP and TADP was investigated at temperatures of 25, 35, 45, and 55 °C. The results are shown in Figure 7. The vertical slopes of the plots for both adsorbents indicate the speedy enhancement in solid phase concentrations of Cu(II) with the increase of liquid phase concentrations from 10 to 50 mg/L. Although, when compared to untreated adsorbents (max 290 mg/g), the H_2_O_2_ treated adsorbents showed better adsorption capabilities (max 360 mg/g). For the graph of initial concentration (C_e_) vs. adsorption capacities (q_e_) of Cu(II) ions at different temperatures, the correlation coefficient (R^2^) ranged from 0.9816 to 0.9926. Higher adsorbate concentration gradients may act as a driving factor for mass transfer between the solution and solid phases to overcome the resistance barrier [39].

The slopes of the plots eventually fell and became nearly parallel to the x-axis, suggesting that the adsorbent surfaces were saturated with Cu(II) ions. The rise in Cu(II) ions adsorption capacity on both ADP and TADP with temperature supports the endothermic adsorption process, and the results are consistent with the literature on metal ion adsorption [44].

Cu(II) ions adsorption capacities on ADP were 60.0–2263.0 mg/g at 298 K, while TADP adsorption capacities (qe) were found to be in the range of 75.3–333.0 mg/g for concentrations ranging from 10 to 50 mg/L. The qe values were increased with increasing temperature and were found to be highest at 318 K for all analyzed concentrations, and the solid phase concentration (q_e_) of Cu(II) ions on ADP were 68.2–290.0 mg/g, while the ranges at temperature 298 K were 60.0–2263.0 mg/g. Similarly, the solid phase concentration on TADP was found to be in the range of 83.2–360.0 mg/g, while the qe were in the ranges of 75.3–333.0 mg/g at temperature 318, and 298 K, respectively. It is obvious that a better adsorption of the target metals was achieved for the treated adsorbent material.

#### 3.2.3. Effects of Contact Time

**Figure 8:**
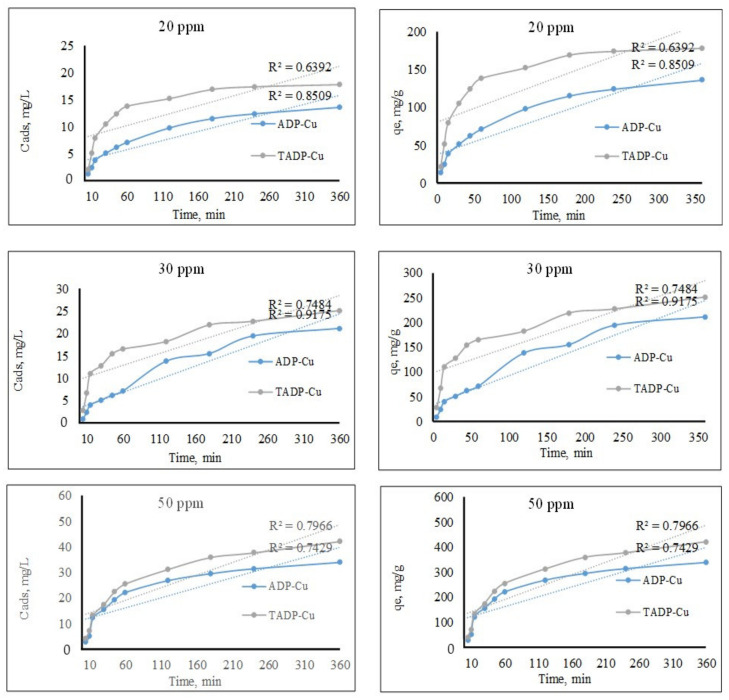
Effect of contact time at concentrations 20, 30, and 50 mg/L of the adsorbates (experimental conditions: Co: 25 mg/L; m: 0.005 g; V: 0.05 L; T: 25 °C; agitation speed: 100 rpm).

Cu(II) ions adsorption on ADP and TADP as a function of contact duration was investigated for Cu(II) concentrations ranging from 5 to 1440 min. Figure 8 shows how contact time (up to 360 min) affects Cu(II) adsorption.

The higher contact time was neglected due to a very minor increase in the adsorption capacities after 360 min. Cu(II) adsorption was detected on ADP and TADP at a slow rate, reaching equilibrium adsorption in 360 min (6 h). For Cu(II) ions adsorption, the correlation coefficient (R^2^) for the graph of time (min) vs. adsorbed concentration (Cads) and Time vs. Qe (mg/g) at concentration levels 20, 30, and 50 mg/L was found to be in the range of 0.6392 to 0.9175. The equilibrium adsorption capacity (Qe) of Cu(II) ions on ADP was 136, 211, and 339 mg/g, for concentrations of 20, 30, and 50 ppm, respectively, and 178, 251, and 421 mg/g for concentrations of 20, 30, and 50 ppm, respectively. The results show that ADP as produced materials have lower adsorption capabilities than TADP.

#### 3.2.4. Adsorption Modeling

##### Equilibrium Isotherm

During the isotherm study, linear isothermal models such as the Langmuir and Freundlich [45] isotherms were applied.

The non-linearized and linearized Langmuir isotherm models are written as follows:

(6) $$q_{e}=\frac{q_{m}k_{L}C_{e}}{1+k_{L}C_{e}},$$

(7) $$\frac{C_{e}}{q_{e}}=\frac{1}{k_{L}q_{m}}+\frac{1}{q_{m}}\;X\;C_{e},$$

where *q_m_* (mg/g) symbolizes maximum monolayer adsorption capacity and *K_L_* (L/mg) denotes the process heat of adsorption.

The Freundlich isotherm, on the other hand, is stated as in [45] in both linear and non-linear models:

(8) $$q_{e}={\;K}_{F}{\;X\;C}_{e}{}^{1/n},$$

(9) $$\log q_{e}=\log K_{F}+\frac{1}{n}\;\log C_{e}.$$

The Freundlich constants for bonding energy and deviation from linearity in adsorption are *K_F_* ((mg/g) (L/mg)^(1/n)^) and *n*, respectively. When the value of *n* in the equation is equal to 1, <1 or >1, the adsorption process is said to be linear, chemical, or physical, respectively.

The adsorption of Cu(II) on ADP and TADP adsorbents was carried out at varied temperatures and the corresponding parameters for adsorption are given in Table 2. The regression coefficient (R^2^) of Langmuir isotherm model for Cu(II) ions adsorption on both ADP and TADP materials at various temperatures was found to be closer to unity than the Freundlich model, which is consistent with previously reported results of metal ion removal [39]. Furthermore, the appropriateness of the Langmuir model shows that monolayer metal ions cover both ADP and TADP adsorbents at various temperatures. The maximum adsorption capacity (*q_m_*) for Cu(II) was found to be increased from 666.67 mg/g (666.67/63.546 = 10.491 mmol/g) to 1428.57 mg/g (1428.57/63.546 = 22.481 mmol/g) for TADP for changing the temperature from 298 K to 328 K. On the other hand, for the same temperature changes the *q_m_* of ADP was changed from 348.0 mg/g (348.0/63.546 = 5.476 mmol/g) to 1111.1 mg/g (1111.1/63.546 = 17.485 mmol/g). The adsorbents and Cu(II) ions may have collided more frequently as the temperature increased, enhancing the adsorption capability of the materials. As the temperature rises, the surface bindings of the adsorbents are ruptured, exposing the active site to a greater extent and therefore increasing the adsorption of the metal ions.

The separation factor (*K_L_*) for Cu(II) ions adsorption on ADP and TADP was in the range of 0.01 to 0.08 at the investigated temperature ranges (298 to 328 K), indicating an adsorption process. The *K_F_* values enhanced as the temperature increased due to the increased contact between metal ions adsorbents, and n > 1 for both adsorbents confirm the physical adsorption process (Table 2).

### 3.3. Adsorption Kinetics

Using the adsorption kinetic data and the models, the Pseudo-first [45] and Pseudo-second-order [45] kinetics models in their linear form were investigated to determine the kinetic parameters. The linearized kinetic models are expressed as follows:

(10) $$\log\left(q_{e1\;}{-\;q}_{t}\right)=\log q_{e1}-\frac{k_{1}}{2.303}X\;t,$$

(11) $$\frac{t}{q_{t}}=\frac{1}{k_{2}q_{e2}{}^{2}}+\frac{1}{q_{e2}}\;X\;t,$$

where *q_e1,_ q_e2,_* and *q_t_* signify the pseudo-first order model, the pseudo-second order model at equilibriums, and the pseudo-third order model at time *t_,_* respectively. Furthermore, *k_1_* and *k_2_* are the rate constants for pseudo-first-order and pseudo-second-order kinetics, respectively.

The obtained kinetic parameters for Cu(II) adsorption data on both ADP and TADP adsorbents are depicted in Table 3. Based on higher R2 values, the Cu(II) ions adsorption data in all tested adsorbents supports a kinetic model with a pseudo-second order (closer to unity). The close agreement between experimental and calculated adsorption capabilities (q_e,exp_) further confirmed the findings, showing that Cu(II) ion adsorption on ADP and TADP was a chemical adsorption process rather than a conventional mass transport phenomenon [46]. Similar findings were described by Trikkaliotis et al. for Cu(II)) adsorption onto chitin based materials [47].

### 3.4. Adsorption Thermodynamics

Van’t Hoff plots [48] are used to estimate the thermodynamic parameters related to Cu(II) ions adsorption on ADP and TADP, such as standard free entropy changes (ΔS°), enthalpy changes (ΔH°), and free energy changes (ΔG°), and the results are shown in Table 4. Cu(II) ions adsorption on both ADP and TADP was endothermic, as demonstrated by positive ΔH° values at all concentrations tested. The ΔS° values for both adsorbents were all positive, suggesting a random adsorption process due to energy redistribution between Cu(II) ions and the adsorbent [49]. Moreover, the ΔG° values for Cu(II) adsorption were negative for all types of adsorbents, suggesting the adsorption process to be spontaneous. The negative ΔG° values rose with an increase in temperature, indicating that the adsorption process favored spontaneity [41].

### 3.5. Elution and Regeneration Studies

**Figure 9:**
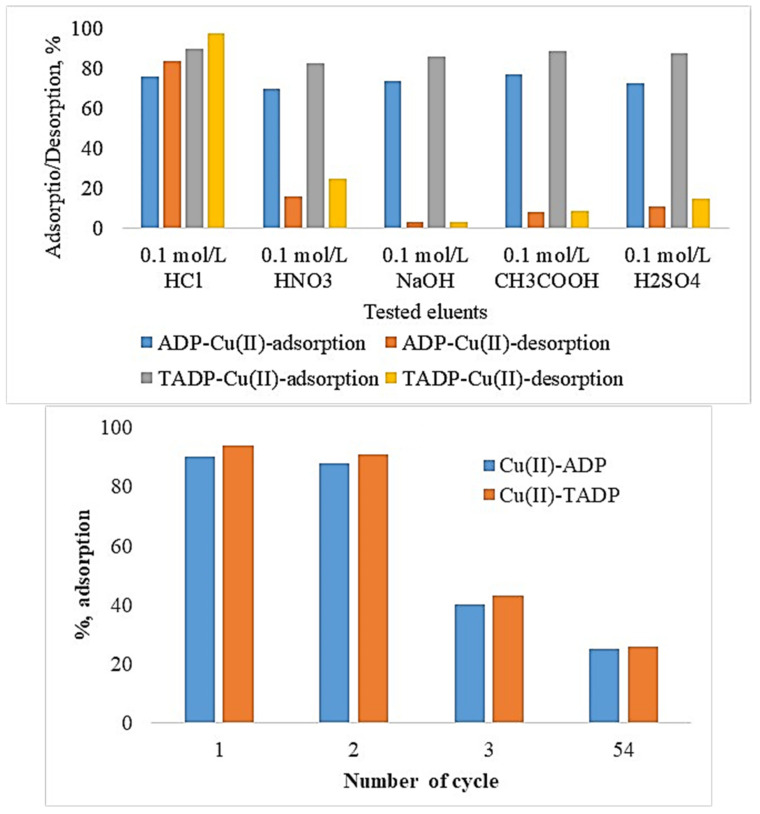
Cu(II) ion elution from saturated adsorbents (experimental conditions: Co: 25 mg/L; m: 0.05 g; V: 0.05 L; T: 25 °C; contact time: 5 h; agitation speed: 100 rpm).

The elution efficiency of sufficiently Cu(II) ions saturated ADP and TADP adsorbents with various eluents was tested (Figure 9). Metal ion elution from both saturated adsorbents was discovered to be modest (3.0–3.2%) when eluted with 0.1 mol/L NaOH, however 0.1 M HCl caused a substantial increase in metal ion desorption for both adsorbents (84–98%).

The order of elution efficacy with the treated eluents was as follows: 0.1 mol/L NaOH < 0.1 mol/L CH_3_COOH < 0.1 mol/L H_2_SO_4_ < 0.1 mol/L HNO_3_ < 0.1 mol/L HCl. The data shows that the highest Cu(II) ion elution with HCl (0.1 mol/L) was 98% for TADP and 84% for ADP. The increased desorption efficacy of Cu(II) ions with relatively strong acid can be attributed to ion-exchange adsorption, which is mediated by metal ions binding on adsorbent surfaces.

After optimizing the eluent (HCl-0.1 mol/L), the regeneration studies for the adsorbents were also carried out to estimate their reusing efficiency. A drastic decrease in Cu(II) (80–89%) adsorption on both ADP and TADP were noticed after the second regeneration investigation. Repeated adsorption-regeneration studies may cause surface morphological deformation, which could be one of the reasons for a decrease in metal ion adsorption with increased regeneration experiments. As a result, ADP and TADP may be repurposed for Cu(II) adsorption with minimal adsorption efficiency loss during the second regeneration.

## 4. Conclusions

The optimized ADP and TADP revealed the excellent adsorption potential of Cu(II) in its respective adsorption methods. The biosorbent TADP showed higher adsorption capabilities compared to ADP. The R^2^ values for C_e_ v.s q_e_ graph at various temperatures for Cu(II) adsorption were found to be 0.9816 to 0.9926, where the R^2^ for time (min) vs. Qe (mg/g) graph at variable concentrations ranged from 0.6392 to 0.9175. The metal ions adsorptions by ADP and TADP were endothermic, indicating that a pseudo-second-order kinetics model would be more appropriate in all circumstances. The Langmuir isotherm model produced somewhat better findings in comparison to the Freundlich model for both ADP and TADP, indicating monolayer coverage. Desorption experiments with 0.1 mol/L HCl yielded the highest Cu(II) ions recovery. The findings of the adsorption and desorption outcomes reveal that the synthesized materials have the potential to be useful for removing heavy metal ions and for other contaminants as well from a variety of media while maintaining a safe environment.

## Figures and Tables

**Table 1:** EDX analysis of the as-prepared ADP and TADP samples.

Element	(keV)	Mass%			
		ADP	Error	TADP	Error
C K	0.277	45.37	0.15	42.96	0.21
N K	0.392	25.60	0.68	21.18	0.45
O K	0.525	29.03	0.18	35.86	0.16
Total		100		100	

**Table 2:** Isotherm parameters for the adsorption of Cu(II) on ADP and TADP.

Isotherm	Temperature, K			
	298	308	318	328
TADP				
Langmuir *q_m_* (mg/g) *K_L_* (L/mg) R^2^	666.67 0.057915 0.9889	909.09 0.01449 0.9972	1111.11 0.01289 0.9986	1428.57 0.01107 0.9932
Freundlich *KF* (mg/g) (L/mg)^1/n^ n R^2^	19.1134 1.2449 0.9096	37.6320 1.2358 0.9253	53.6320 1.2858 0.9170	57.0176 1.4550 0.9250
ADP				
Langmuir *q_m_* (mg/g) *K_L_* (L/mg) R^2^	348.0 0.03275 0.9853	625.0 0.07048 0.9901	769.2 0.01289 0.9932	1111.1 0.03904 0.9891
Freundlich *KF* (mg/g) (L/mg)^1/n^ n R^2^	18.52 1.0132 0.7192	23.43 1.2168 0.8586	23.69 1.6211 0.8914	48.56 1.7621 0.8979

**Table 3:** Kinetic parameters calculated for the adsorption of Cu(II) on ADP and TADP adsorbents (initial concentration, C_0_ for Cu(II) was 20 mg/L).

Kinetics Model	ADP	TADP
	**Cu(II) 20 mg/L**	
q_e,exp_ (mg/g)	136	178
Pseudo-first-order		
q_e1,cal_ (mg/g)	118.38	126.88
K_1_(1/min)	0.0097	0.0145
R^2^	0.991	0.978
Pseudo-second-order		
q_e2,cal_ (mg/g)	145.25	189.07
k_2_(g/mg-min)	0.00005	0.00003
R^2^	0.996	0.995

**Table 4:** Thermodynamic parameters for the adsorption of Cu(II) on ADP and TADP adsorbents (initial concentration, C_0_ was 20 mg/L).

Adsorbents	Concentration mg/L	ΔH°	ΔS°	ΔG° (kJ/mol)			
		(kJ/mol)	(J/mol-K)	298 K	308 K	318 K	328 K
ADP	20	2608.34	86.29	−156.34	−566.44	−951.43	−1020.48
	30	2122.10	69.92	−165.79	−341.35	−708.64	−916.92
	40	1810.70	60.54	−419.21	−479.90	−562.61	−821.86
	50	1762.03	59.16	−64.62	−92.97	−423.11	−634.68
TADP	20	1017.01	41.52	−2167.69	−2537.27	−2934.88	−3064.55
	30	704.95	28.99	−1533.09	−1904.93	−2098.57	−2178.40
	40	854.00	34.71	−1786.02	−2073.66	−2380.42	−2550.16
	50	896.01	30.83	−257.90	−409.23	−899.72	−1004.94
