# Correction: Azam et al. Heavy Metal Ions Removal from Aqueous Solutions by Treated Ajwa Date Pits: Kinetic, Isotherm, and Thermodynamic Approach. *Polymers* 2022, *14*, 914

**DOI:** 10.3390/polym16152121

**Published:** 2024-07-25

**Authors:** Mohammad Azam, Saikh Mohammad Wabaidur, Mohammad Rizwan Khan, Saud I. Al-Resayes, Mohammad Shahidul Islam

**Affiliations:** Department of Chemistry, College of Science, King Saud University, P.O. Box 2455, Riyadh 11451, Saudi Arabia; swabaidur@ksu.edu.sa (S.M.W.); sresayes@ksu.edu.sa (S.I.A.-R.); mislam@ksu.edu.sa (M.S.I.)

In the original publication, there was a mistake in Figure 2B as the wrong image was uploaded. The corrected SEM images of ADP (A–C) and TADP (D–F) at various magnifications—1000× with a 10 μm diameter; 3000× with a 5 μm diameter; and 6000× with a 2 μm diameter—appear below. The authors state that the scientific conclusions are unaffected. This correction was approved by the Academic Editor. The original publication has also been updated [1].

## Figures and Tables

**Figure 2:**
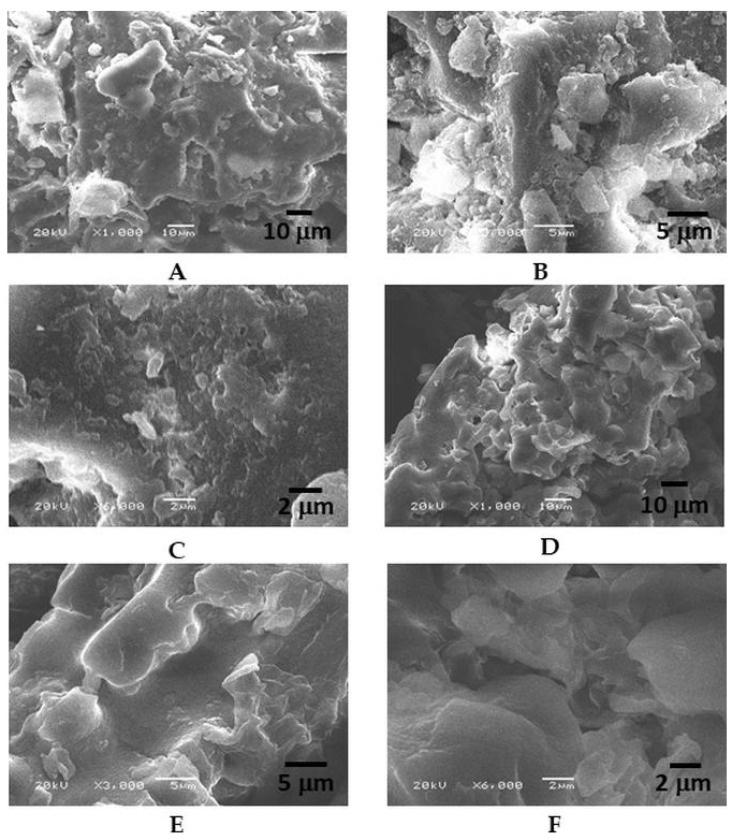
SEM images of ADP (**A**–**C**) and TADP (**D**–**F**) at various magnification: 1000× and 10 μm; 3000× and 5 μm: and 6000× magnification with 2 μm diameter.
